# Atypical multidrug-resistant serotype 1 *Actinobacillus pleuropneumoniae* with *ApxIII* in China

**DOI:** 10.3389/fvets.2026.1849275

**Published:** 2026-07-01

**Authors:** Chuchu Duan, Zhihong Fang, Zecheng Lin, Xiaojin Liu, Zhongfeng Luo, Ying Wang, Yudi Liu, Ling Li, Cuiqin Huang, Zehua Jin, Zuchen Song, Xiaohua Li, Xintian Zheng

**Affiliations:** 1 College of Animal Science, Fujian Agriculture and Forestry University, Fuzhou, Fujian, China; 2 College of Life Sciences, Longyan University, Longyan, Fujian, China; 3 Fujian Provincial Key Laboratory of Animal Infectious Disease Prevention and Biotechnology, Longyan, Fujian, China

**Keywords:** *Actinobacillus pleuropneumoniae*, *ApxIII*, atypical, drug resistance, pathogenicity

## Abstract

*Actinobacillus pleuropneumoniae* (APP) is the primary etiological agent of porcine contagious pleuropneumonia and is responsible for substantial economic losses in the swine industry worldwide. In April 2023, an acute respiratory disease outbreak occurred on a pig farm in Longyan City, Fujian Province, China, resulting in a morbidity rate of 30% and a mortality rate of 56%. This study aimed to isolate and identify the causative pathogen and to characterize its major biological properties. An NAD-dependent bacterial strain was isolated from the lung tissues of deceased pigs and identified as APP serotype 1 through morphological characterization, biochemical testing, and serotype-specific PCR analysis. The isolate was designated APPFJLYC01. Toxin gene profiling revealed the presence of the *ApxII*, *ApxIII*, and *ApxIV* genes, whereas the *ApxI* gene was absent. This toxin gene pattern differs from the typical profile reported for serotype 1 strains (*ApxI*, *ApxII*, and *ApxIV*), suggesting potential genetic diversity in the distribution of toxin-associated genes among local APP populations. Antimicrobial susceptibility testing demonstrated that APPFJLYC01 was resistant to polymyxin, tetracycline, enrofloxacin, ampicillin, gentamicin, and amoxicillin/clavulanic acid, indicating a multidrug-resistant phenotype. Pathogenicity assessment in mice confirmed the virulence of the isolate, with a median lethal dose (LD_50_) of 7.91 × 10^8^ CFU/mL. In conclusion, this study reports the isolation and characterization of a serotype 1 APP strain from Longyan, Fujian Province, exhibiting an atypical toxin gene profile and multidrug resistance. These findings provide valuable epidemiological data for understanding the genetic diversity and antimicrobial resistance characteristics of APP strains circulating in the region and may contribute to the development of effective prevention and control strategies for porcine pleuropneumonia.

## 1 Introduction

*Actinobacillus pleuropneumoniae* (APP) is the causative agent of porcine contagious pleuropneumonia, a severe respiratory disease that causes substantial economic losses in the swine industry (1–3). Based on surface antigens and nicotinamide adenine dinucleotide (NAD) dependency, APP is classified into 19 serotypes and two biotypes (4, 5). Cross-protection among serotypes is limited, and their distribution varies geographically (6). In China, serotypes 1, 3, 5, and 7 are the predominant serotypes, with serotype 5, 7 becoming increasingly prevalent in recent years (3).

The virulence of APP is primarily associated with its RTX toxins, including *ApxI*, *ApxII, ApxIII,* and *ApxIV. ApxI* possesses strong hemolytic and cytotoxic activities, *ApxII* exhibits weaker toxicity, and *ApxIII* is highly cytotoxic but lacks hemolytic activity. *ApxIV* is conserved among all APP serotypes and is commonly used as a species-specific molecular marker for APP detection (7–9). The production of biologically active *Apx* toxins requires both the structural gene *ApxA* and the activator gene *ApxC*, whereas *ApxB* and *ApxD* are responsible for toxin secretion (10). Recent studies have leveraged this knowledge to develop a septuple-gene deletion live attenuated vaccine by targeting multiple *Apx* toxin-associated genes, including *ApxIC, ApxIIC*, and *ApxIV* (11). Previous studies have demonstrated that *ApxI* is the most virulent RTX toxin, followed by *ApxIII*, whereas *ApxII* exhibits the lowest virulence among the three toxins (12). The distribution of *Apx* toxin genes is strongly associated with APP serotypes. For example, serotype 1 strains typically harbor the *ApxICABD, ApxIICA*, and *ApxIVA* gene clusters, encoding *ApxI, ApxII*, and *ApxIV*, respectively (13). However, recent investigations have revealed variations in toxin gene profiles among certain APP isolates (14), suggesting that genetic diversity within toxin-associated genes may contribute to differences in virulence and pose challenges for disease prevention and control.

Due to the extensive serotype diversity of APP and the limited cross-protection conferred by current vaccines, antimicrobial therapy remains a cornerstone for the prevention and control of porcine contagious pleuropneumonia (PCP). However, resistance to commonly used antimicrobial agents, particularly tetracyclines and sulfonamides, has become increasingly prevalent (15, 16). A large-scale epidemiological survey conducted in China between 2021 and 2024 revealed a marked increase in the proportion of multidrug-resistant (MDR) APP isolates, rising from 0% in 2021 to 42.1% in 2024 (17). Similarly, in Taiwan, China, 71.4% of clinical isolates exhibited multidrug resistance, with resistance genes associated with aminoglycosides and tetracyclines being the most prevalent (18). Consistent with these findings, a retrospective study in Italy reported that approximately one-third of APP isolates were resistant to three or more classes of antimicrobial agents (19).

In April 2023, an acute respiratory disease outbreak occurred among 70-day-old piglets on a commercial pig farm in Longyan, Fujian Province, China, causing a morbidity rate of 30% and a mortality rate of 56%. Despite treatment with florfenicol and tilmicosin, the outbreak remained uncontrolled. In our previous study, whole-genome sequencing of APPFJLYC01 revealed the presence of 107 strain-specific genes and 190 virulence factor homologs. Furthermore, its hemolysin toxin genes exhibited substantial genetic divergence compared with those of other reference strains, showing sequence identities ranging from 42.4 to 86.1% (20). This study aimed to isolate and identify the causative pathogen and to characterize its serotype, toxin gene profile, antimicrobial resistance phenotype, and pathogenicity. The results provide epidemiological data for APP surveillance in Fujian Province and offer a basis for disease control and future genomic studies of circulating APP strains.

## 2 Materials and methods

### 2.1 Clinical samples and experimental animals

Lung and bronchial tissue samples were aseptically collected from pigs that died during a suspected *Actinobacillus pleuropneumoniae* (APP) outbreak on a commercial pig farm in Longyan, Fujian Province, China.

Sixty BALB/c mice (20 ± 2 g) were purchased from Jiangsu Speyford (Suzhou) Biotechnology Co., Ltd. (Certificate No. 202401822). All animal experiments were approved by the Animal Ethics Committee of Longyan University (Approval No. LY2024014L) and were conducted in accordance with the Guidelines for the Welfare and Ethics of Laboratory Animals.

At the conclusion of the experiment, mice that survived were humanely euthanized by cervical dislocation under deep anesthesia induced by intraperitoneal injection of tribromoethanol (300 mg/kg). All procedures were performed in accordance with institutional animal welfare guidelines, and every effort was made to minimize animal suffering. Death was confirmed by the absence of vital signs.

### 2.2 Culture media and major reagents

General agar, Columbia blood agar base, tryptic soy agar (TSA), and tryptic soy broth (TSB) were purchased from Qingdao Hope Bio-Technology Co., Ltd. (Qingdao, China). Chocolate agar, Salmonella–Shigella (SS) agar, and MacConkey agar were obtained from Beijing Land Bridge Technology Co., Ltd. (Beijing, China). Nicotinamide adenine dinucleotide (NAD) was purchased from Beijing Solarbio Science & Technology Co., Ltd. (Beijing, China). The DL2000 DNA Marker was obtained from Takara Biomedical Technology (Beijing) Co., Ltd. (Beijing, China). The 2 × Taq Master Mix was purchased from Vazyme Biotech Co., Ltd. (Nanjing, China), and nucleic acid stain was supplied by Sangon Biotech Co., Ltd. (Shanghai, China). Miniaturized biochemical identification tubes were obtained from Hangzhou Tianhe Microbial Reagent Co., Ltd. (Hangzhou, China). Specific item numbers are listed in Supplementary Table S1.

### 2.3 Bacterial isolation and cultivation

Lung and bronchial tissue samples collected from deceased pigs were aseptically streaked onto tryptic soy agar (TSA) supplemented with 5% fetal bovine serum and 0.5% nicotinamide adenine dinucleotide (NAD), followed by incubation at 37 °C under microaerophilic conditions using a candle jar for 24–48 h. Individual colonies were selected and purified by subculture. The purified isolates were subsequently inoculated onto sheep blood agar supplemented with 0.5% NAD, MacConkey agar, and TSA plates for further characterization.

### 2.4 Morphological and biochemical identification

Purified isolates were subjected to Gram staining and examined microscopically for morphological characterization. Biochemical properties were determined using miniaturized biochemical identification tubes containing 14 biochemical tests, including glucose fermentation, maltose fermentation, and urease activity. The inoculated tubes were incubated at 37 °C for 24–48 h, and the results were interpreted according to the manufacturer’s instructions and standard biochemical characteristics of *Actinobacillus pleuropneumoniae*.

### 2.5 PCR identification based on the 16S rRNA gene

Genomic DNA was extracted from purified isolates using the boiling method and used as the template for PCR amplification. Primers targeting the 16S rRNA gene were designed and synthesized (Supplementary Table S2). PCR was performed with an initial denaturation at 95 °C for 5 min, followed by 35 cycles of denaturation at 95 °C for 30 s, annealing at 55 °C for 45 s, and extension at 72 °C for 1 min, with a final extension at 72 °C for 10 min. PCR products were analyzed by agarose gel electrophoresis, and amplicons of the expected size were sequenced by Sangon Biotech Co., Ltd. (Shanghai, China). The obtained sequences were analyzed using BLAST against the NCBI database for species identification.

### 2.6 Serotyping and toxin gene detection

Serotyping was performed by PCR using 19 pairs of serotype-specific primers targeting APP serotypes 1–19, as previously described by Stringer et al. (6). Genomic DNA from the isolate was used as the template for amplification. PCR products were analyzed by agarose gel electrophoresis, and positive amplicons were sequenced by Sangon Biotech Co., Ltd. (Shanghai, China). The obtained sequences were compared with reference APP sequences available in the GenBank database.

Toxin gene detection was conducted using specific primers designed with Primer Premier 5 software (Supplementary Table S2). PCR amplification was performed using genomic DNA as the template, and the amplified products were analyzed by agarose gel electrophoresis.

To confirm the atypical *Apx* gene profile identified by PCR, comparative genomic analyses were performed using the complete *ApxI* and *ApxIII* reference sequences. The complete *ApxI* sequence (GenBank accession no. X68595.1, 8,292 bp) was retrieved from NCBI and aligned with the APPFJLYC01 genome to assess the presence of the *apxIA, ApxIBD*, and *ApxIC* regions. Similarly, the complete *ApxIII* sequence (GenBank accession no. X80055.1, 15,746 bp) was used to evaluate the integrity of the *ApxIII* in APPFJLYC01.

### 2.7 Phylogenetic analysis

To investigate the genetic relationships between the isolate and representative APP serotypes, 14 APP reference sequences were retrieved from the GenBank database (Table 1). Multiple sequence alignment was performed using MAFFT software (21), followed by sequence trimming. A phylogenetic tree was constructed using the Neighbor-Joining (NJ) method implemented in MEGA 6.0, based on the Kimura two-parameter (K2P) model, with 1,000 bootstrap replicates to assess branch support (22).

**Table 1:** Strains and genomic sequences used in this study.

Strain	Serovar	Accession number
APPFJLYC01	Serovar 1	CP182859.1
4074	Serovar 1	CP029003.1
KL16	Serovar 1	CP022715.1
P1875	Serovar 2	CP079921.1.
JL03	Serovar 3	CP000687.1
APP5	Serovar 5	CP063424.1
L20	Serovar 5b	CP000569.1
APP6	Serovar 6	CP026009.1
AP76	Serovar 7	CP001091.1
WF83	Serovar 7	CP031869.1
S405	Serovar 8	CP078508.1
MIDG2331	Serovar 8	LN908249.1
CVJ13261	Serovar 9	CP031865.1
17-039	Serovar 12	CP190096.1
A85/14	Serovar 15	CP069795.1

### 2.8 Antimicrobial susceptibility testing

Antimicrobial susceptibility was determined by the broth microdilution method as previously described (20, 23–26). The test medium was supplemented with 0.5% nicotinamide adenine dinucleotide (NAD) and 5% fetal bovine serum. Serial two-fold dilutions of each antimicrobial agent were prepared at concentrations ranging from 0.125 to 512 μg/mL. Following inoculation, the plates were incubated aerobically at 37 °C for 24 h. A total of 10 antimicrobial agents representing different classes were evaluated, including florfenicol (amphenicol), cefotaxime (cephalosporin), polymyxin B (polypeptide), tilmicosin (macrolide), doxycycline and tetracycline (tetracyclines), enrofloxacin (fluoroquinolone), ampicillin (penicillin), gentamicin (aminoglycoside), and amoxicillin/clavulanic acid (β-lactam/β-lactamase inhibitor combination). MIC values were interpreted according to the Clinical and Laboratory Standards Institute (CLSI) guidelines.

### 2.9 Pathogenicity assay

Sixty BALB/c mice were randomly assigned to six groups (n = 10 per group). Mice in Groups 1–5 were intraperitoneally inoculated with 1 mL of bacterial suspension containing 2.5 × 10^10^, 2.5 × 10^9^, 2.5 × 10^8^, 2.5 × 10^7^, and 2.5 × 10^6^ CFU/mL, respectively. Mice in Group 6 received 1 mL of sterile physiological saline and served as the negative control. Following inoculation, mice were monitored daily for clinical signs and mortality. The median lethal dose (LD_50_) was calculated using the Reed–Muench method. Dead animals were subjected to necropsy, and gross pathological lesions were recorded. Mice exhibiting severe clinical signs were humanely euthanized in accordance with the approved animal ethics protocol.

### 2.10 Histopathological examination

At the end of the experiment, surviving mice were humanely euthanized and subjected to necropsy. Tissue samples from the heart, liver, spleen, lungs, and kidneys were collected and fixed in 10% neutral-buffered formalin for 24 h. The tissues were then processed routinely, embedded in paraffin, sectioned, and stained with hematoxylin and eosin (H&E). Histopathological changes were examined and photographed under a light microscope.

## 3 Results

### 3.1 Isolation and morphological characterization of the isolate

The isolate grew well on modified TSA plates supplemented with 5% fetal bovine serum and 0.5% NAD, as well as on NAD-supplemented sheep blood agar plates. No bacterial growth was observed on TSA plates lacking NAD supplementation. In addition, no hemolytic activity was detected on sheep blood agar.

**Figure 1:**
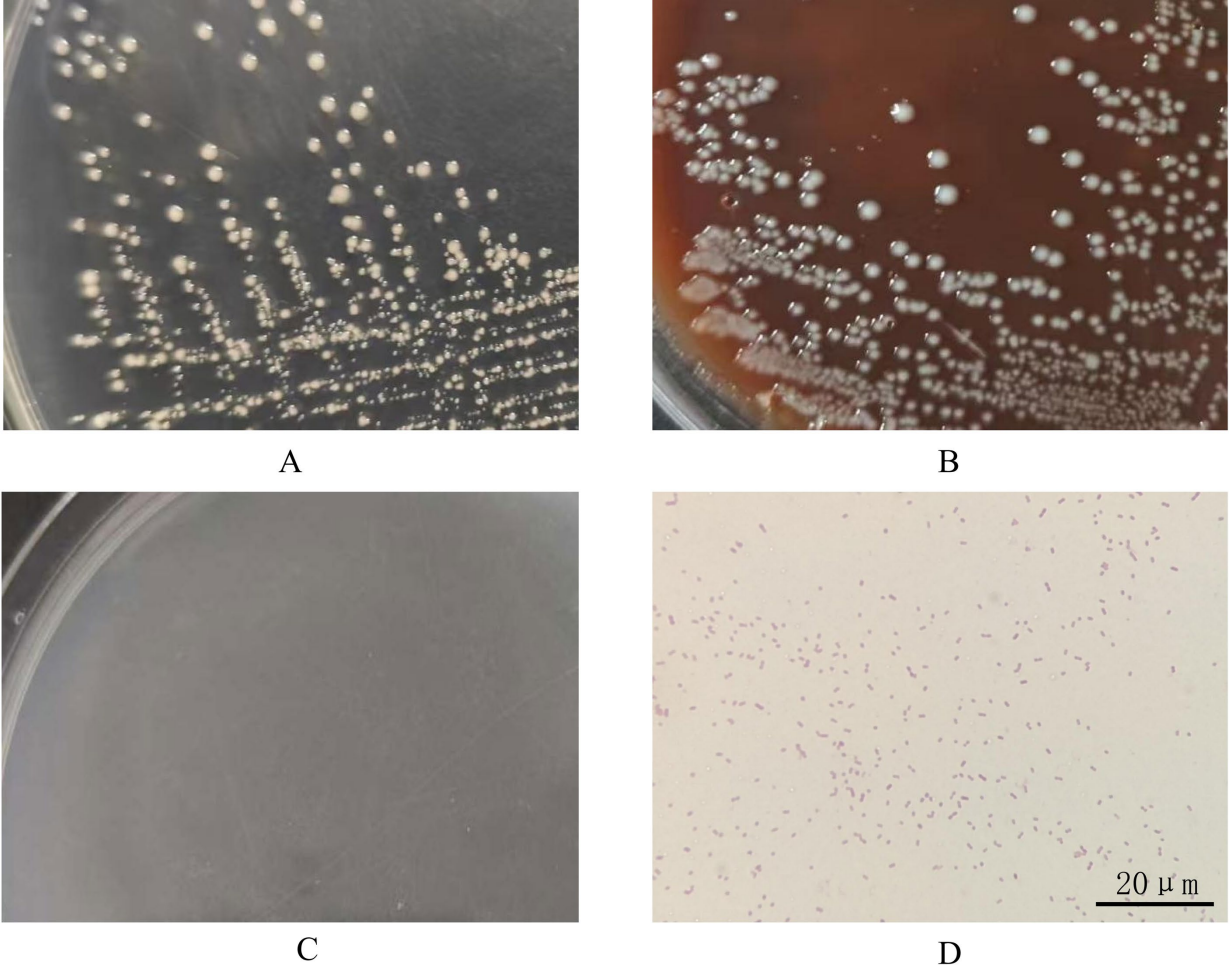
Colony morphology and Gram staining characteristics of the isolate. Growth on modified tryptic soy agar (TSA) supplemented with 5% fetal bovine serum and 0.5% NAD **(A)**, growth on NAD-supplemented sheep blood agar **(B)**, absence of growth on TSA without NAD supplementation **(C)**, and Gram staining showing Gram-negative short rod-shaped bacteria **(D)**. Scale bar = 20 μm.

The colonies displayed typical characteristics of biotype I APP, appearing circular, smooth, moist, translucent, and non-pigmented with regular margins. Gram staining showed that the isolate was Gram-negative and predominantly composed of short rod-shaped bacteria under light microscopy (Figure 1).

### 3.2 Biochemical identification

The biochemical characteristics of the isolate are summarized in Table 2. The strain fermented glucose, maltose, and sucrose and exhibited positive urease activity, while sorbitol fermentation, mannitol fermentation, and H₂S production were negative. Overall, the biochemical profile was consistent with that of *Actinobacillus pleuropneumoniae.*

**Table 2:** Biochemical identification results of the isolated strain.

Item	Result	Item	Result
Glucose	+	Ornithine	−
Maltose	+	Phenylalanine	−
Lactose	−	Urease	+
Sucrose	+	Gluconate	−
Mannitol	−	Citrate	−
Sorbitol	−	H_₂_S	−
Lysine	−		

+, Positive; −, Negative.

### 3.3 16S rRNA gene amplification and identification

**Figure 2:**
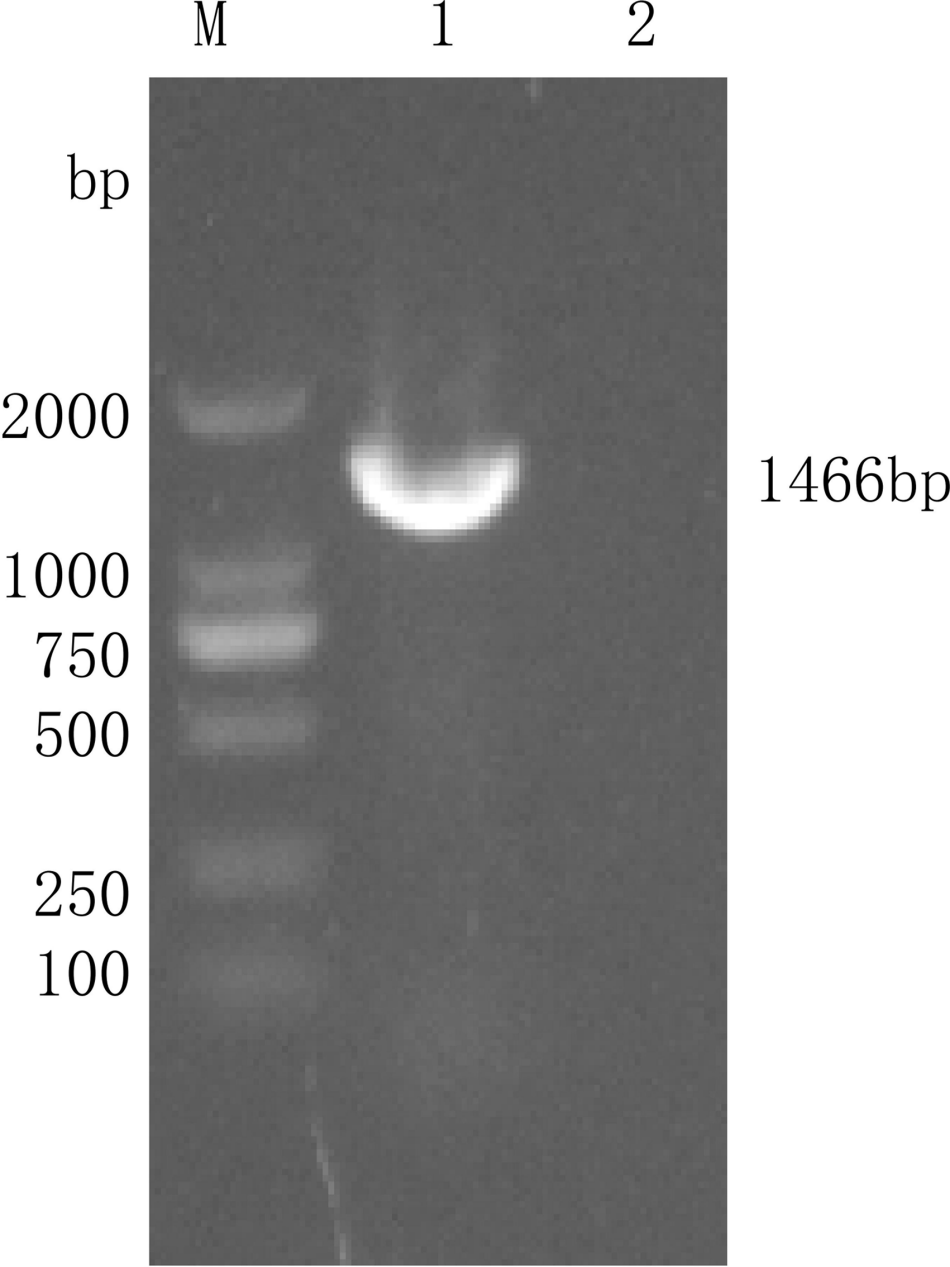
PCR amplification of the 16S rRNA gene from the isolate. Lane M: DL2000 DNA marker; Lane 1: 16S rRNA PCR product (approximately 1,466 bp); Lane 2: negative control.

Amplification of the 16S rRNA gene generated an amplicon of approximately 1,466 bp (Figure 2). BLAST analysis of the obtained sequence revealed ≥99% identity with APP sequences in the GenBank database, confirming that the isolate belonged to *A. pleuropneumoniae*.

### 3.4 Serotype identification and toxin gene detection

**Figure 3:**
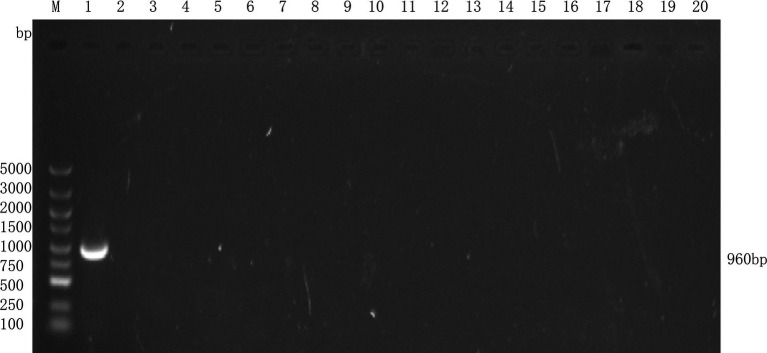
PCR-based serotype identification of the APP isolate. Lane M: DL5000 DNA marker; Lanes 1–19: PCR products amplified using serotype-specific primers for APP serotypes 1–19, respectively. A specific amplicon was detected only in Lane 1, indicating that the isolate belongs to APP serotype 1. Lane 20: negative control.

PCR-based serotyping was performed using serotype-specific primers targeting APP serotypes 1–19. As shown in Figure 3, a specific amplicon of approximately 960 bp was detected only with the serotype 1 primer set, whereas no amplification was observed with primers targeting other serotypes. These results identified the isolate as APP serotype 1, designated APPFJLYC01.

**Figure 4:**
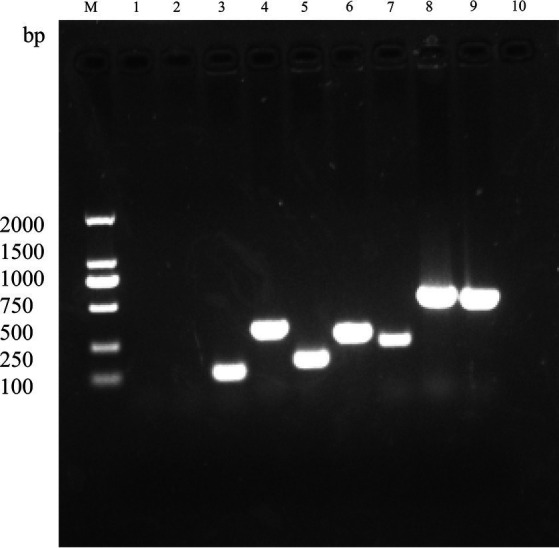
PCR detection of *Apx* toxin-associated genes in APPFJLYC01. Lane M: DL2000 DNA marker; Lane 1: *ApxIA* (723 bp); Lane 2: *ApxIC* (131 bp); Lane 3: *ApxIBD* (117 bp); Lane 4: *ApxIIA* (358 bp); Lane 5: *ApxIIC* (168 bp); Lane 6: *ApxIIIA* (320 bp); Lane 7: *ApxIIIC* (250 bp); Lane 8: *ApxIIIBD* (570 bp); Lane 9: *ApxIV* (530 bp); Lane 10: negative control. No amplification was observed in Lanes 1 and 2, indicating the absence of *ApxIA* and *ApxIC*, whereas the remaining target genes were successfully amplified.

Toxin gene profiling revealed that APPFJLYC01 lacked the *ApxIA* (723 bp) and *ApxIC* (131 bp) genes but carried *ApxIBD* (117 bp), *ApxIIA* (358 bp), *ApxIIC* (168 bp), *ApxIIIA* (320 bp), *ApxIIIC* (250 bp), *ApxIIIBD* (570 bp), and *ApxIV* (530 bp), as confirmed by PCR amplification (Figure 4). These results indicate that APPFJLYC01 possesses the genetic determinants required for the production and secretion of *ApxII*, *ApxIII*, and *ApxIV* toxins.

Comparative genomic analysis confirmed the deletion of *ApxIA* and *ApxIC* while retaining the complete *ApxIBD* (1,616,174–1,611,539 bp) region in APPFJLYC01. In contrast, the APPFJLYC01 genomic region spanning positions 1,539,914–1,532,045 bp corresponded to the complete *ApxIII* locus and showed 99.898% sequence coverage relative to the reference sequence X80055.1, indicating that the *ApxIII* gene cluster was intact. These results verified the atypical *Apx* gene profile detected by PCR. Detailed sequence comparison results are presented in Supplementary Figures S1–S4.

### 3.5 Phylogenetic analysis

**Figure 5:**
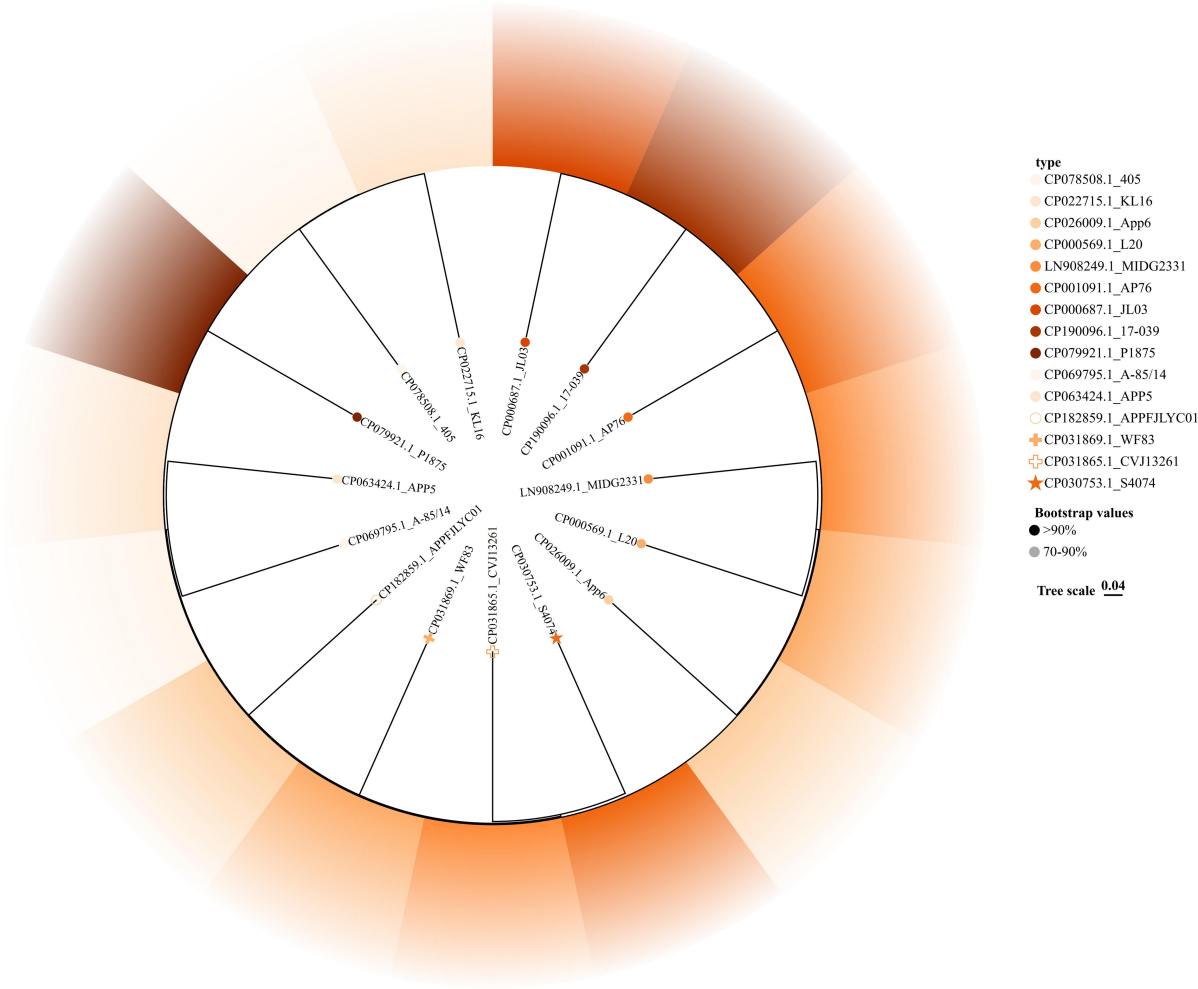
Neighbor-Joining phylogenetic tree. Showing the relationship between APPFJLYC01 and 14 reference *Actinobacillus pleuropneumoniae* (APP) strains based on whole-genome sequence analysis. The tree was constructed using the Neighbor-Joining method with 1,000 bootstrap replicates. Branch lengths indicate genetic divergence among strains, and bootstrap values are shown at the nodes. Strain names and corresponding GenBank accession numbers are displayed adjacent to the inner branches. Different strains within the ring have different colors.

Phylogenetic analysis revealed that APPFJLYC01 was closely related to strains WF83, S4074, and CVJ13261. APPFJLYC01 clustered within the same lineage as these strains but formed an independent branch supported by a bootstrap value of 98. 4,074 and CVJ13261 constituted the closest subcluster (bootstrap value = 100). Other reference strains formed distinct phylogenetic groups, including the A-85/14–APP5 cluster and the L20–MIDG2331–App6 cluster. Overall, the major terminal nodes were supported by high bootstrap values, indicating reliable phylogenetic clustering (Figure 5).

### 3.6 Antimicrobial susceptibility testing

The antimicrobial susceptibility results are summarized in Table 3. APPFJLYC01 was susceptible to florfenicol, tilmicosin, and cefotaxime. In contrast, the isolate exhibited resistance to polymyxin B, tetracycline, enrofloxacin, ampicillin, gentamicin, and amoxicillin/clavulanic acid, indicating a multidrug-resistant phenotype.

**Table 3:** Partial antimicrobial susceptibility test results of the isolated strain.

Antimicrobial	Standard (R > μg/mL)	Result	Susceptibility
Florfenicol	>1	1	S
Cefotaxime	>32	4	S
Polymyxin B	>0.5	16	R
Tilmicosin	>32	8	S
Doxycycline	>2	4	R
Tetracycline	>2	64	R
Enrofloxacin	>0.125	1	R
Ampicillin	>0.5	4	R
Gentamicin	>8	32	R
Amoxicillin/Clavulan	>1	4	R

S, susceptible; R, resistant.

The minimum inhibitory concentration (MIC) values of the quality control strain ATCC 27090 were within the quality control ranges recommended by the Clinical and Laboratory Standards Institute (CLSI). All MIC assays were performed in three independent biological replicates.

### 3.7 Pathogenicity analysis

Following challenge with different concentrations of APPFJLYC01, mice in the infected groups developed clinical signs as early as 12 h post-inoculation. The main manifestations included ruffled fur, lethargy, reduced feed intake, tachypnea, and convulsions, with the most severe symptoms observed in Groups 1 and 2. Necropsy of dead mice revealed ascites and marked hemorrhagic lesions in the heart, lungs, and spleen.

**Figure 6:**
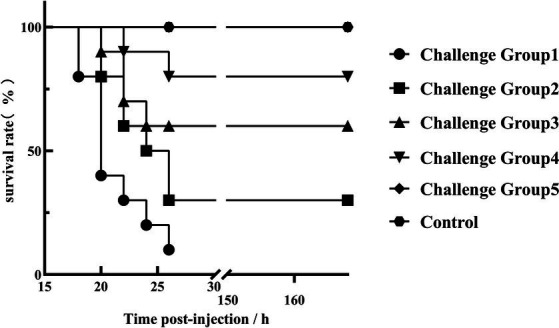
Kaplan–Meier survival curves of mice challenged with different doses of APPFJLYC01. The x-axis is truncated between 30 and 150 h (indicated by a double slash) to omit the interval during which no mortality occurred. Control, uninfected negative control group; Groups 1–5, mice challenged with APPFJLYC01 at different inoculation doses. The survival curve was recorded over the full 0–168 h observation period.

The mortality results are summarized in Table 4. Within 7 days post-challenge, mortality rates decreased with decreasing bacterial dose. Nearly all mice in Group 1 died, whereas 7, 4, and 2 mice died in Groups 2, 3, and 4, respectively. No mortality was observed in Group 5 or the control group (Figure 6; Table 4). Based on the Reed–Muench method, the median lethal dose (LD_50_) of APPFJLYC01 in mice was calculated to be 7.91 × 10^8^ CFU/mL.

**Table 4:** Mortality statistics of mice in each group.

Group	Number of mice	Inoculation dose (CFU/mL)	Inoculation route	Number of deaths	Mortality rate (%)
Challenge Group 1	10	2.5 × 10^10^	Intraperitoneal injection	9	90
Challenge Group 2	10	2.5 × 10^9^	Intraperitoneal injection	7	70
Challenge Group 3	10	2.5 × 10^8^	Intraperitoneal injection	4	40
Challenge Group 4	10	2.5 × 10^7^	Intraperitoneal injection	2	20
Challenge Group 5	10	2.5 × 10^6^	Intraperitoneal injection	0	0
Control Group	10	0	Intraperitoneal injection	0	0

**Figure 7:**
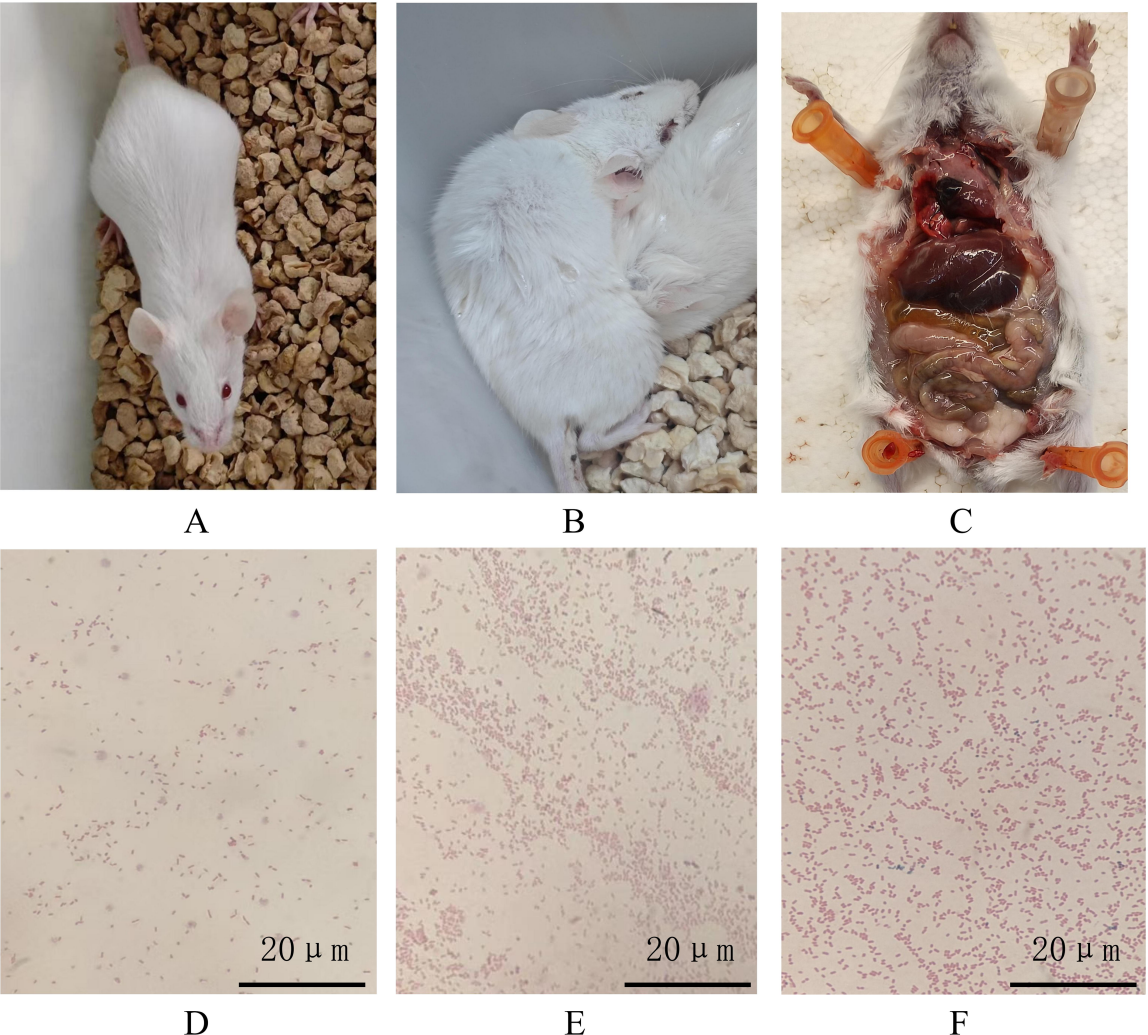
Clinical appearance, necropsy findings, and bacterial reisolation from mice following APPFJLYC01 challenge. **(A)** Mouse from the uninfected control group; **(B)** mouse from Challenge Group 1; **(C)** representative gross lesions observed during necropsy; **(D)** bacterial reisolation from lung tissue; **(E)** bacterial reisolation from thoracic exudate; and **(F)** bacterial reisolation from peritoneal exudate. Gram staining of the reisolated bacteria revealed Gram-negative short rod-shaped organisms consistent with APPFJLYC01. Scale bars = 20 μm **(D–F)**.

Lung tissue, thoracic exudate, and peritoneal exudate collected from mice that died in Group 1 were subjected to bacterial reisolation (Figure 7). The recovered isolates exhibited colony morphology identical to that of APPFJLYC01. Gram staining revealed Gram-negative short rod-shaped bacteria, and PCR amplification of the 16S rRNA gene confirmed that the recovered isolates shared the same molecular characteristics as APPFJLYC01, demonstrating successful reisolation of the challenge strain.

### 3.8 Histopathological analysis

**Figure 8:**
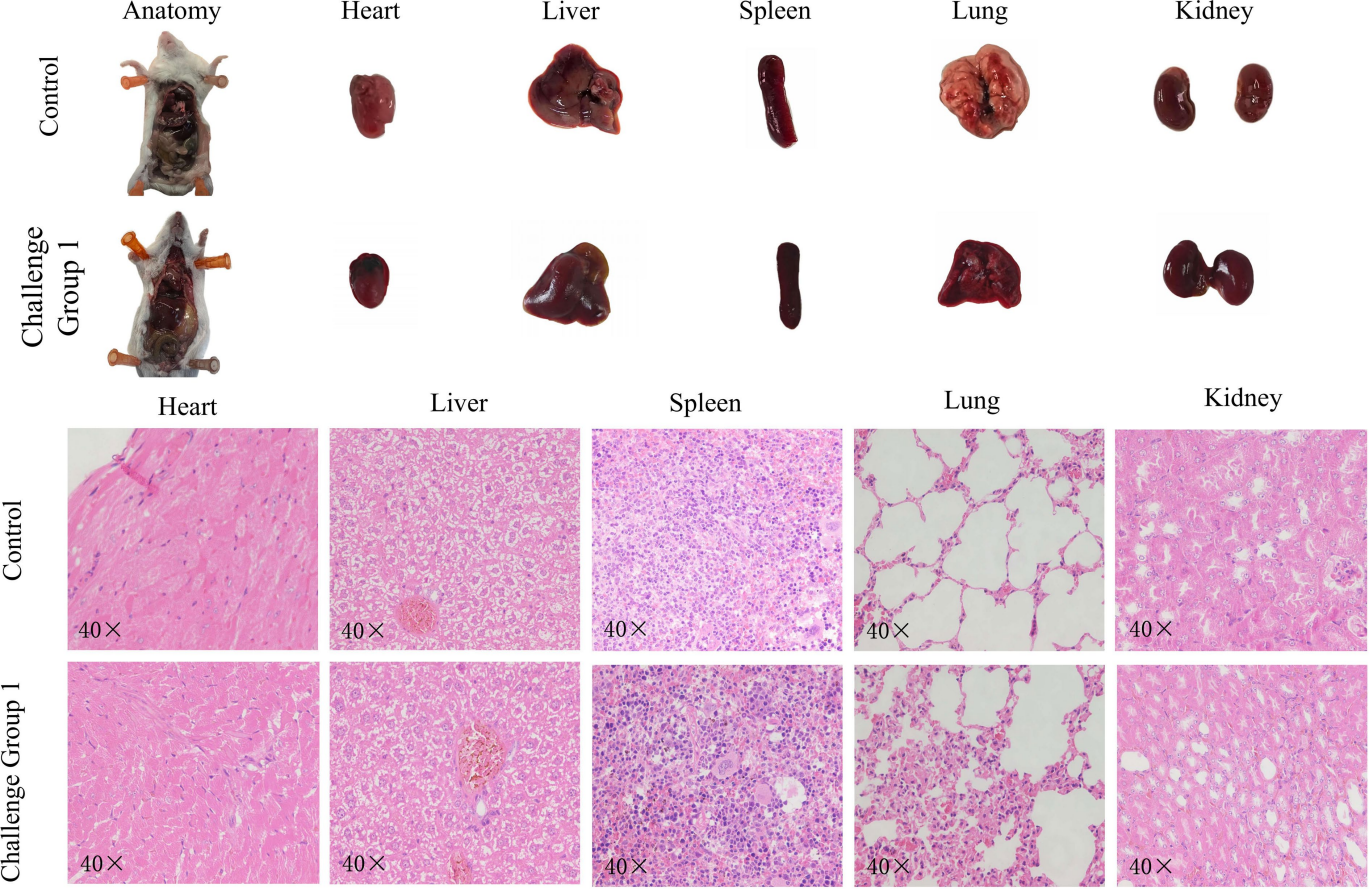
Gross lesions and histopathological changes in mice following APPFJLYC01 challenge. The upper panels show representative gross pathological lesions observed in challenged mice, including whole-body appearance and lesions in the heart, liver, spleen, lungs, and kidneys. The lower panels show corresponding hematoxylin and eosin (H&E)-stained sections from challenged and control mice. Histopathological images were captured at 40 × magnification.

Histopathological examination was performed on the heart, liver, spleen, lungs, and kidneys collected from mice in Challenge Group 1 and the control group (Figure 8). Compared with the control group, mice challenged with APPFJLYC01 exhibited marked pathological lesions in multiple organs.

Cardiac tissue showed myocardial interstitial edema, widening of the fibrous septa, and mild inflammatory cell infiltration. In the liver, focal hemorrhage, hepatocellular degeneration and edema, disruption of hepatic lobular architecture, and nuclear fading were observed. Splenic lesions were characterized by severe inflammatory cell infiltration and increased hemosiderin deposition. Pulmonary lesions included destruction of alveolar architecture, bronchial epithelial damage with thickening of the bronchial wall, congestion of alveolar capillaries, and interstitial inflammatory cell infiltration. Renal lesions consisted of vacuolar degeneration of the renal tubular epithelium and focal glomerular cell hyperplasia.

## 4 Discussion

Currently, acute outbreaks of porcine contagious pleuropneumonia (PCP) are causing significant economic losses worldwide due to high mortality. APP exists globally, but the prevalence of serotypes differs by region. Currently, the dominant circulating serotypes in China are primarily 1, 3, 5, and 7 (3).

Serotype 1 APP strains typically harbor *ApxI, ApxII*, and *ApxIV* (*ApxICABD, ApxIICA, ApxIV*). APPFJLYC01, however, carried an atypical profile (*ApxII, ApxIII, ApxIV*), suggesting ongoing genetic diversification in local populations. Such toxin gene variations may result from horizontal gene transfer (HGT), recombination, or regional evolution (27). Prophages and mobile elements facilitate HGT and genome recombination, while intensive farming and intergeneric genetic exchange further promote toxin gene diversification (28–32). Similar atypical strains with altered toxin genes or capsule-associated mutations have been reported globally, indicating these variants are not geographically restricted (29, 33–35). APP pathogenicity depends on the coordinated action of *Apx* toxins, which differ in cytotoxic activity and contribute to colonization, immune evasion, and tissue damage (11, 36–39). In APPFJLYC01, the absence of *ApxIA* and *ApxIC* with presence of *ApxIII* suggests a distinct toxin repertoire. Whole-genome sequence analysis confirmed the absence of *ApxIA* and *ApxIC* while retaining the complete *ApxIBD* region and the entire *ApxIII* operon (*ApxIIIC, ApxIIIA, ApxIIIBD*). However, the presence or absence of toxin-associated genes does not necessarily reflect toxin activity, as RTX-family *Apx* toxins require *ApxC*-mediated post-translational acylation for activation (40–42). Therefore, alterations in toxin expression, activation, or secretion may influence virulence independently of gene content. Since protein expression and functional characterization were not performed in the present study, the biological significance of this atypical toxin profile remains unclear. Elucidating the evolutionary origin and functional consequences of this genetic arrangement will be the focus of future investigations.

Antimicrobial susceptibility testing demonstrated that APPFJLYC01 exhibited resistance to polymyxin B, doxycycline, tetracycline, enrofloxacin, ampicillin, gentamicin, and amoxicillin/clavulanate, indicating a multidrug-resistant phenotype. Similar resistance patterns have been increasingly reported worldwide. Recent surveillance studies documented doxycycline resistance rates of 77.8% in China and 32.8% in Poland, while tetracycline resistance reached 53% in Italy (18, 20, 43). In Taiwan, resistance to amoxicillin and other commonly used veterinary antimicrobials has also been reported (44). These findings, together with our whole-genome analysis identifying resistance determinants associated with β-lactams, tetracyclines, aminoglycosides, and macrolides (20), indicate that APPFJLYC01 belongs to the growing population of multidrug-resistant APP strains. This trend highlights the importance of antimicrobial susceptibility testing and prudent antimicrobial use in clinical practice.

Mouse challenge experiments demonstrated that APPFJLYC01 was capable of causing lethal infection, with an LD_50_ of 7.91 × 10^8^ CFU/mL. This value is substantially higher than those reported for highly virulent APP strains, whose LD₅₀ values typically range from 10^4^ to 10^6^ CFU/mL (45, 46), indicating relatively low virulence in the mouse model. A possible explanation is the absence of *ApxIA* and *ApxIC*, which prevents production of functional *ApxI* toxin, a major virulence determinant of highly pathogenic serotype 1 strains. Similar associations between toxin gene mutations and reduced virulence have been reported previously (11, 35, 39, 47). Although APPFJLYC01 retained the ability to induce mortality and characteristic pathological lesions in mice, its virulence appears lower than that of classical highly virulent serotype 1 strains. The limitations of the animal model: Because mice are not the natural host of APP, LD50 values obtained in this model may not fully reflect the isolate’s pathogenicity in swine. Further studies in swine and at the protein-expression level are required to clarify the contribution of the atypical toxin gene profile to pathogenicity.

In conclusion, APPFJLYC01 represents a serotype 1 APP strain characterized by an atypical toxin gene profile, multidrug resistance, and relatively low virulence in mice. These findings expand current knowledge regarding the genetic and phenotypic diversity of APP field isolates and highlight the importance of continuous molecular surveillance for emerging variants with altered virulence or antimicrobial resistance characteristics.

## 5 Conclusion

This study identified and characterized a pathogenic serotype 1 APP strain from Fujian Province, China, with an atypical toxin gene profile and a multidrug-resistant phenotype. The results provide new insights into the genetic diversity, virulence potential, and antimicrobial resistance of APP field strains and underscore the importance of continuous surveillance to guide disease prevention and targeted antimicrobial therapy.

## Data Availability

The datasets presented in this study can be found in online repositories. The names of the repository/repositories and accession number(s) can be found in the article/Supplementary material.

## References

[1] HuY JiangC ZhaoY CaoH RenJ ZengW . TurboID screening of *ApxI* toxin interactants identifies host proteins involved in *Actinobacillus pleuropneumoniae*-induced apoptosis of immortalized porcine alveolar macrophages. Vet Res. (2023) 54:62. doi: 10.1186/s13567-023-01194-6, 37475032 PMC10360236

[2] Tobias TJ Klinkenberg D Bouma A van den Broek J Daemen AJJM Wagenaar JA . A cohort study on *Actinobacillus pleuropneumoniae* colonisation in suckling piglets. Prev Vet Med. (2014) 114:223–30. doi: 10.1016/j.prevetmed.2014.02.008, 24630401

[3] Sassu EL Bossé JT Tobias TJ Gottschalk M Langford PR Hennig-Pauka I. Update on *Actinobacillus pleuropneumoniae*—knowledge, gaps and challenges. Transbound Emerg Dis. (2018) 65:72–90. doi: 10.1111/tbed.12739, 29083117

[4] ZhouY LiL ChenZ WurteleES ZhangD LuC . Outer surface protein C peptide derived from *Borrelia burgdorferi* sensu stricto as a target for serodiagnosis of early Lyme disease. Clin Vaccine Immunol. (2013) 20:474–81. doi: 10.1128/CVI.00608-12, 23365204 PMC3623407

[5] BosséJT LiY SárköziR FodorL LacoutureS GottschalkM . Antimicrobial susceptibility of enterococci recovered from healthy cattle, pigs and chickens in nine EU countries (EASSA study) to critically important antibiotics. Vet Microbiol. (2018) 216:168–75. doi: 10.1016/j.vetmic.2018.02.010, 29519512

[6] Stringer OW Bossé JT Lacouture S Gottschalk M Fodor L Angen Ø . Proposal of *Actinobacillus pleuropneumoniae* serovar 19, and reformulation of previous multiplex PCRs for capsule-specific typing of all known serovars. Vet Microbiol. (2021) 255:109021. doi: 10.1016/j.vetmic.2021.109021, 33667982

[7] Tascón RI Vázquez-Boland JA Gutiérrez-Martín CB Rodríguez-Barbosa JI Rodríguez-Ferri EF. The RTX hemolysin *ApxI* and *ApxII* are major virulence factors of the swine pathogen *Actinobacillus pleuropneumoniae*: evidence from mutational analysis. Mol Microbiol. (1994) 14:207–16. doi: 10.1111/j.1365-2958.1994.tb01282.x, 7830567

[8] Frey J Bossé JT Chang YF Cullen JM Fenwick B Gerlach GF . *Actinobacillus pleuropneumoniae* RTX-toxins: uniform designation of hemolysins, cytolysins, pleurotoxin and their genes. J Gen Microbiol. (1993) 139:1723–8. doi: 10.1099/00221287-139-8-1723 8409915

[9] Stancheva SG Frömbling J Sassu EL Hennig-Pauka I Ladinig A Gerner W . Proteomic and immunoproteomic insights into the exoproteome of *Actinobacillus pleuropneumoniae*, the causative agent of porcine pleuropneumonia. Microb Pathog. (2022) 172:105759. doi: 10.1016/j.micpath.2022.105759, 36087692

[10] GuoF QuanR CuiY CaoX WenT XuF. Effects of OxyR regulator on oxidative stress, Apx toxin secretion and virulence of *Actinobacillus pleuropneumoniae*. Front Cell Infect Microbiol. (2023) 13:1324760. doi: 10.3389/fcimb.2023.1324760, 38268788 PMC10806198

[11] Cao W He Q Chen Y Liu J Wei Z Wei X . Development and assessment of a septuple-deletion mutant live attenuated vaccine for *Actinobacillus pleuropneumoniae* in swine. Pak Vet J. (2024) 44:1229–36. doi: 10.29261/pakvetj/2024.297

[12] MaierE ReinhardN BenzR FreyJ. Channel-forming activity and channel size of the RTX toxins *ApxI*, *ApxII*, and *ApxIII* of *Actinobacillus pleuropneumoniae*. Infect Immun. (1996) 64:4415–23. doi: 10.1128/IAI.64.11.4415-4423.1996, 8890186 PMC174392

[13] SoutterF PriestnallSL CatchpoleB WattegederaSR SturrockCJ HuntleyJF . An experimental dermal oedema model for Apx toxins of *Actinobacillus pleuropneumoniae*. J Comp Pathol. (2022) 195:12–8. doi: 10.1016/j.jcpa.2022.08.00235817536

[14] KampEM VermeulenTM SmitsMA HaagsmaJ den HartighJ FroymannR. Production of Apx toxins by field strains of *Actinobacillus pleuropneumoniae* and *Actinobacillus suis*. Infect Immun. (1994) 62:4063–5. doi: 10.1128/IAI.62.9.4063-4065.1994, 8063425 PMC303069

[15] WangYC ChanJP YehKS ChangCC HsuYM ChenTH . The increase in seroprevalence of bluetongue virus (BTV) serotype 8 infections and associated risk factors in Dutch dairy herds, in 2007. Vet Microbiol. (2010) 142:268–75. doi: 10.1016/j.vetmic.2009.10.026, 19945231

[16] MaX ZhengB WangJ LiG CaoS WenY . Quinolone resistance of *Actinobacillus pleuropneumoniae* revealed through genome and transcriptome analyses. Int J Mol Sci. (2021) 22:10036. doi: 10.3390/ijms221810036, 34576206 PMC8472844

[17] Peng L Yang D Han W Chen H Zhang J Zhou Q . Epidemiological and antimicrobial resistance characteristics of recent (2021–2024) Chinese *Actinobacillus pleuropneumoniae* clinical isolates. Anim Dis. (2006) 6:27. doi: 10.1186/s44149-026-00237-7

[18] KeCH LaiPY HsuFY LinWH ChenCM TsaiYL . Antimicrobial susceptibility and resistome of *Actinobacillus pleuropneumoniae* in Taiwan: a next-generation sequencing analysis. Vet Q. (2024) 44:1–13. doi: 10.1080/01652176.2024.2306376PMC1106473638688482

[19] GuarneriF RomeoC ScaliF FerrarioCR TonniM MerialdiG . Serotype diversity and antimicrobial susceptibility profiles of *Actinobacillus pleuropneumoniae* isolated in Italian pig farms from 2015 to 2022. Vet Res. (2024) 55:48. doi: 10.1186/s13567-024-01305-x, 38594744 PMC11005290

[20] FangZ LinZ DuanC LiuX LuoZ HuangC . Genome analysis of *Actinobacillus pleuropneumoniae* strain APPFJLYC01 reveals multidrug resistance and high virulence potential. PLoS One. (2025) 20:e0336060. doi: 10.1371/journal.pone.0336060, 41237104 PMC12617859

[21] VandenbusscheF MathijsE TignonM CayAB NauwynckHJ MaesD . WGS- versus ORF5-based typing of PRRSV: a Belgian case study. Viruses. (2021) 13:2419. doi: 10.3390/v13122419, 34960688 PMC8707199

[22] TohH KatohK. Parallelization of the MAFFT multiple sequence alignment program. Bioinformatics. (2010) 26:1899–900. doi: 10.1093/bioinformatics/btq224, 20427515 PMC2905546

[23] GutiérrezCB del BlancoM NavasJ Rodríguez-FerriEF. Changes in antimicrobial susceptibility of *Actinobacillus pleuropneumoniae* isolated from pigs in Spain during the last decade. Vet Microbiol. (2007) 115:218–22. doi: 10.1016/j.vetmic.2006.12.009, 16431040

[24] ArchambaultM HarelJ GoureJ TremblayYDN JacquesM. Antimicrobial susceptibilities and resistance genes of Canadian isolates of *Actinobacillus pleuropneumoniae*. Microb Drug Resist. (2012) 18:198–206. doi: 10.1089/mdr.2011.0149, 22204596

[25] Matter D Rossano A Limat S Vorlet-Fawer L Brodard I Perreten V. Antimicrobial resistance profile of Actinobacillus pleuropneumoniae and Actinobacillus porcitonsillarum. Vet Microbiol. (2007) 122:146–56. doi: 10.1016/j.vetmic.2007.01.009, 17314014

[26] de Jong A Thomas V Simjee S Moyaert H El Garch F Maher K . Antimicrobial susceptibility monitoring of respiratory tract pathogens isolated from diseased cattle and pigs across Europe: the VetPath study. Vet Microbiol. (2014) 172:202–15. doi: 10.1016/j.vetmic.2014.04.008, 24837878

[27] DonàV RoschanskiN VötschD SchwendenerS PerretenV FreyJ . Comparative genomics of 26 complete circular genomes of 18 different serotypes of *Actinobacillus pleuropneumoniae*. Microb Genom. (2022) 8:000776. doi: 10.1099/mgen.0.000776, 35196217 PMC8942016

[28] PradoIGO SilvaGC CrispimJS de OliveiraNR dos SantosLF RossiCC . Comparative genomics of *Actinobacillus pleuropneumoniae* serotype 8 reveals the importance of prophages in the genetic variability of the species. Int J Genomics. (2020) 2020:9354204. doi: 10.1155/2020/9354204, 32149072 PMC7049842

[29] dos SantosLF SinceroTCM de SouzaTA SilvaGC RossiCC. Polymorphism analysis of the ApxIA gene of *Actinobacillus pleuropneumoniae* serovar 5 isolated in swine herds from Brazil. PLoS One. (2018) 13:e0208789. doi: 10.1371/journal.pone.0208789, 30562362 PMC6298653

[30] XuZF ChenX LiL ZhouR YueJ WuC . Genome-wide evidence for positive selection and recombination in *Actinobacillus pleuropneumoniae*. BMC Evol Biol. (2011) 11:203. doi: 10.1186/1471-2148-11-203, 21749728 PMC3146884

[31] TegetmeyerHE JonesSCP LangfordPR BaltesN. ISApl1, a novel insertion element of *Actinobacillus pleuropneumoniae*, prevents *ApxIV*-based serological detection of serotype 7 strain AP76. Vet Microbiol. (2008) 128:342–53. doi: 10.1016/j.vetmic.2007.10.017, 18065168

[32] WattAE BrowningGF LegioneAR DevlinJM MichalopoulouE SansomFM . A novel *Glaesserella* sp. isolated from pigs with severe respiratory infections has a mosaic genome with virulence factors putatively acquired by horizontal transfer. Appl Environ Microbiol. (2018) 84:e00092-18. doi: 10.1128/AEM.00092-18, 29572210 PMC5960975

[33] Lindhaus H Bischoff H Harms M Schwendener S Frey J Donà V . Comparison of molecular serotyping methods for *Actinobacillus pleuropneumoniae* and analysis of atypical serotypes detected in routine diagnostics. J Microbiol Methods. (2025) 232:107132. doi: 10.1016/j.mimet.2025.107132, 40245988

[34] VincentAT LacoutureS St- JeanG AugerE DaignaultD GagnonCA . Atypical *Actinobacillus pleuropneumoniae* serotype 12 strains with a higher virulence potential. Vet Res. (2025) 56:149. doi: 10.1186/s13567-025-01571-740653462 PMC12255999

[35] ToH KobayashiK NakaiT GottschalkM FreyJ BosséJT . Characterization of atypical *Actinobacillus pleuropneumoniae* serovar 1 isolates. J Vet Diagn Invest. (2026) 38:259–64. doi: 10.1177/1040638725141288541563096 PMC12823378

[36] Soto- PerezchicaMM Guerrero- BarreraAL Avelar- GonzalezFJ Mora- MendozaDA Ramos- AragonJA VacaS . *Actinobacillus pleuropneumoniae*, surface proteins and virulence: a review. Front Vet Sci. (2023) 10:1276712. doi: 10.3389/fvets.2023.1276712, 38098987 PMC10720984

[37] ToH FreyJ GottschalkM BosséJT SassuEL Hennig- PaukaI . Lack of evidence for transmission of atypical H‐type bovine spongiform encephalopathy prions (H‐BSE prions) by intracranial and oral challenges to nonhuman primates. Microbiol Immunol. (2025) 69:25–34. doi: 10.1111/1348-0421.13180, 39523908

[38] Slivenecka E Jurnecka D Holubova J Kralova M Osicka R Sebo P . The *Actinobacillus pleuropneumoniae* ApxIV operon encodes an antibacterial toxin-immunity pair. Microbiol Res. (2025) 292:128043. doi: 10.1016/j.micres.2024.128043, 39740637

[39] RamjeetM CoxAD HancockMA MourezM LabrieJ JacquesM. Mutation in the LPS outer core biosynthesis gene galU affects LPS interaction with the RTX toxins *ApxI* and *ApxII* and cytolytic activity of *Actinobacillus pleuropneumoniae* serotype 1. Mol Microbiol. (2008) 70:221–35. doi: 10.1111/j.1365-2958.2008.06405.x, 18713318

[40] GreeneNP CrowA HughesC KoronakisV. Structure of a bacterial toxin-activating acyltransferase. Proc Natl Acad Sci USA. (2015) 112:6431–6. doi: 10.1073/pnas.1504725112, 26016525 PMC4466738

[41] O'BrienDP DurandES VoegeleA HourdelV DaviM Chamot-RookeJ . The nuclear concentration required for antisense oligonucleotide activity in myotonic dystrophy cells. FASEB J. (2019) 33:11314–25. doi: 10.1096/fj.201900263R, 31311315

[42] Tzfira T. The comprehensive sourcebook of bacterial protein toxins. Third edition. By Joseph E Alouf and Michel R popoff. Academic press. Burlington (Massachusetts): Elsevier. $199.95. Xxiii + 1047 p; ill.; index. ISBN: 0‐12‐088445‐3. 2006. Q Rev Biol. (2007) 82:50–11. doi: 10.1086/513350

[43] PrzyborowskaP DorsA PejsakZ Pomorska-MólM. Serotyping and antimicrobial resistance of *Actinobacillus pleuropneumoniae* isolates from fattening pigs in Poland from 2019 to 2024. BMC Vet Res. (2019) 21:40. doi: 10.1186/s12917-025-04339-2PMC1177627739881342

[44] KwanWF LinYL JengCR TsaiYL ChenCM HsuFY . Serovars and antimicrobial resistance profiles of *Actinobacillus pleuropneumoniae* isolates from clinical-case pigs in Taiwan. BMC Vet Res. (2025) 21:502. doi: 10.1186/s12917-025-04601-740759949 PMC12323125

[45] KomalJPS MittalKR. Grouping of *Actinobacillus pleuropneumoniae* strains of serotype 1 through serotype 12 on the basis of their virulence in mice. Vet Microbiol. (1990) 25:229–40. doi: 10.1016/0378-1135(90)90085-S, 2281607

[46] Sebunya TNK Saunders JR. Studies on immunity to *Haemophilus pleuropneumoniae* infections in mice. Am J Vet Res. (1982) 43:1793–8. doi: 10.2460/ajvr.1982.43.10.1793, 7149378

[47] Nascimento KA Pereira DA Santos ACR Silva N Oliveira LG Costa MM . Updates on mechanisms of action, diagnosis and control of *Actinobacillus pleuropneumoniae* in pig infections. Arch Zootec. (2016) 65:623–9.

