# Supplementary material for: Atypical multidrug-resistant serotype 1 Actinobacillus pleuropneumoniae with ApxIII in China
Source: Front Vet Sci. 2026 Jul 1;13:1849275. doi: 10.3389/fvets.2026.1849275 (PMC13383674; doi:10.3389/fvets.2026.1849275)
Supplement: Supplementary file 1 [file Supplementary_file_1.docx]

**Supplementary Material**

**Atypical Multidrug-Resistant Serotype 1 *Actinobacillus pleuropneumoniae* with ApxIII in China**

**Chuchu Duan^1,2^, Zhihong Fang^2^, Zecheng Lin^2^, Xiaojin Liu^2^, Zhongfeng Luo^2^, Ying Wang^2^, Yudi Liu^2^,Ling Li^2^, Cuiqin Huang^2,3^, Zehua Jin^2,3^, Zuchen Song^2,3*^, Xiaohua Li^2,3*^,Xintian Zheng^2,3*^**

1. College of Animal Science, Fujian Agriculture and Forestry University, Fuzhou, Fujian China,

2. College of Life Sciences, Longyan University, Longyan, Fujian China,

3. Fujian Provincial Key Laboratory of Animal Infectious Disease Prevention and Biotechnology, Longyan, Fujian China,

* Correspondence and reprint requests:Xintian Zheng, Xiaohua Li, Zuchen Song. College of Life Sciences, Longyan University, Longyan, Fujian China. E-mail:[xintianzheng@lyun.edu.cn](mailto:xintianzheng@lyun.edu.cn) (Xintian Zheng), [1340398694@qq.com](mailto:1340398694@qq.com) (Xiaohua Li), [s1271967668@163.com](mailto:s1271967668@163.com) (Zuchen Song).

**Table S1.**

| Major Reagents | manufacturer | catalog number |
| --- | --- | --- |
| General Agar | Qingdao Haibo Co., Ltd. | HB0128 |
| Columbia Nlood Agar Base | Qingdao Haibo Co., Ltd. | HB0124-15 |
| Tryptic Soy Agar (TSA) | Qingdao Haibo Co., Ltd. | HB7026-9 |
| Tryptic Soy Broth (TSB) | Qingdao Haibo Co., Ltd. | HB4114 |
| Chocolate Agar | Beijing Land Bridge Technology Co., Ltd. | PB001 |
| SS Agar | Beijing Land Bridge Technology Co., Ltd. | CM206 |
| MacConkey Agar | Beijing Land Bridge Technology Co., Ltd. | CM908 |
| Nicotinamide Adenine Dinucleotide (NAD) | Beijing Solarbio Science & Technology  Co., Ltd. | N8110 |
| DL2000 DNA Marker | Takara Biomedical Technology (Beijing) Co., Ltd. | 3427A |
| 2× Taq Master Mix | Nanjing Vazyme Biotech Co., Ltd. | P222-01 |
| Nucleic Acid Stain | Sangon Biotech (Shanghai) Co., Ltd. | A616696-010 |
| Miniaturized biochemical identification tubes | Hangzhou Tianhe Microbial Reagent Co., Ltd. | J2002 |

**Table S2.**

Table S2 Primer Information

| Gene | Primer Sequence (5'→3') | | **Reference /Accession number** |
| --- | --- | --- | --- |
| APP-1 | F:CTGGAGTAATTACGGCGACTATTCC | R:AGGAGAAGCTAGTAGTACTTGCATTTTC | Proposal of Actinobacillus pleuropneumoniae serovar 19, and reformulation of previous multiplex PCRs for capsule-specific typing of all known serovars[J]. Veterinary microbiology, 2021,Vol.255:109021.doi:10.1016/j.vetmic.2021.109021 |
| APP-2 | F:GAGTGTGATGATGATGCTCTGGTTC | R:TACCAATAACTGTTGCAACTAACGC |  |
| APP-3 | F:TTGTAGAGCCCGCCAGATTTACG | R:CATTCGCACCAGCAATCACC |  |
| APP-4 | F:CAGCATGGGTTTGGTCCTGTTG | R:GGCTTTCTCCGTGTATGAATAAAGTG |  |
| APP-5 | F:AGCCACAAGACCCGAATGGTATAATG | R:CCATCAAATGCAGCTTCAAGGAGC |  |
| APP-6 | F:TGACTGGCTTCGTGAAAATGAG | R:GTCTGAAGTTTTATTCGCAGCTCC |  |
| APP-7 | F:TTGGAATGGATTCATGATTGGGC | R:CGGAAATGGCCTATTGAAAAACG |  |
| APP-8 | F:ACATCCAAGCCGTTCTCCAG | R:CATCCATGAGCCAATGAGGG |  |
| APP-9/11 | F:GTAGGACGTGGTAAAACATTGAGGC | R:ACGGGTGCAATTTCTAAAGCTG |  |
| APP-10 | F:GGTGGTGATGGAACAAGGTTATGG | R:CTGTAATTGATGCGAAATAGTAGATTGGTGC |  |
| APP-12 | F:TAAAGGTATTATAACGCCGGCTCT | R:CTCCCATCTGTTGTCTAAGTAGTAG |  |
| APP-13 | F:GTTGTGTATCGAGGTTGGCATTTC | R:ATGTAAAGGATCTAAGCCGTGTG |  |
| APP-14 | F:TGCATTACGCTTATATTCTGAATGG | R:TTGTCGATCGAGAGGGAGTAACG |  |
| APP-15 | F:GCAACTTGGAGAACATGGTTAAATCAAG | R:CAACCCTCCAATGTAAGCGAAGG |  |
| APP-16 | F:TTACTCACTTGGGCTAGGGATAG | R:ACCAGCAATATGATTACGCCC |  |
| APP-17 | F:TTGTAATGGCGGTGTAATGCTAC | R:CATAAGTGCAGCCATCTCTTTCAG |  |
| APP-18 | F:CGGAGTTTGGCAGCATAAAGG | R:CCATAATCGGTGCTCAACTAAGAATG |  |
| APP-19 | F:ATACGGCAAATAATCGAGTTACTGC | R:CTTGAATAACGACCGATAGATACTCC |  |
| 16Sr RNA | F:AGAGTTTGATCCTGGCTCAG | R:GGTTACCTTGTTACGACTT |  |
| *Apx*ⅠA | F:gcgccggtagcagctttagttag | R:taccggtttgcttacgctcacgaa | NZ_CP009159.1 |
| *Apx*ⅠC | F:GTGCTACCTGCGATTGAG | R:ACTAGCGAGGCAACATCA | NZ_CP009159.1 |
| *Apx*ⅠBD | F:AATAGCCAAGCGGTTAGA | R:CGGATTATACGCACTGAC | NZ_CP030753.1 |
| *Apx*ⅡA | F:ttgcgttactcgttgctggtgtta | R:gactggtgttgcccctcctca | LC034182.1 |
| *Apx*ⅡC | F:GAGTGAGGCTCGCTATGT | R:ATTCTAATTGCCCTTCCA | NZ_CP009159.1 |
| *Apx*ⅢA | F:agaagccaaaagacaagccaaaaa | R:gcggtgcaaataaagtaagtcca | LC033890.1 |
| *Apx*ⅢC | F:CCGTTACATAAGGAGTGG | R:AAAGCGTATTGTTATCCC | L12145.1 |
| *Apx*ⅢBD | F:GAAGAACGAAACCCTAAA | R:TGGGCTGTAATACCACAT | L12145.1 |
| *Apx*ⅣA | F:GGAATGGAAAGAAGGGCTAT | R:CACGAGTTATGCTGCGGTC | KX594329.1 |

**Figure.S1**


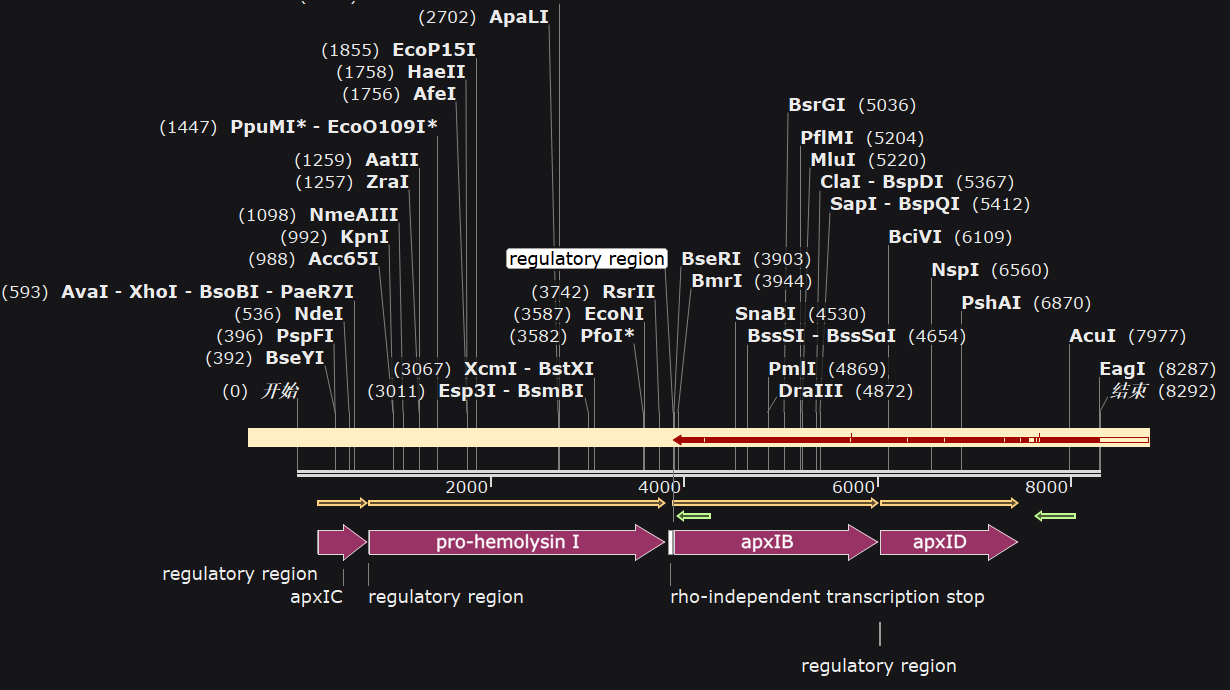


Figure S1. Sequence comparison between X68595.1 and APPFJLYC01.

The sequence shown corresponds to X68595.1, which contains the complete *ApxICABD* gene cluster. The red arrow indicates the region exhibiting sequence homology with APPFJLYC01. This homologous region corresponds to the *ApxIBD* genes, whereas the *ApxIA* and *ApxIC* genes are absent in APPFJLYC01.

**Figure.S2**


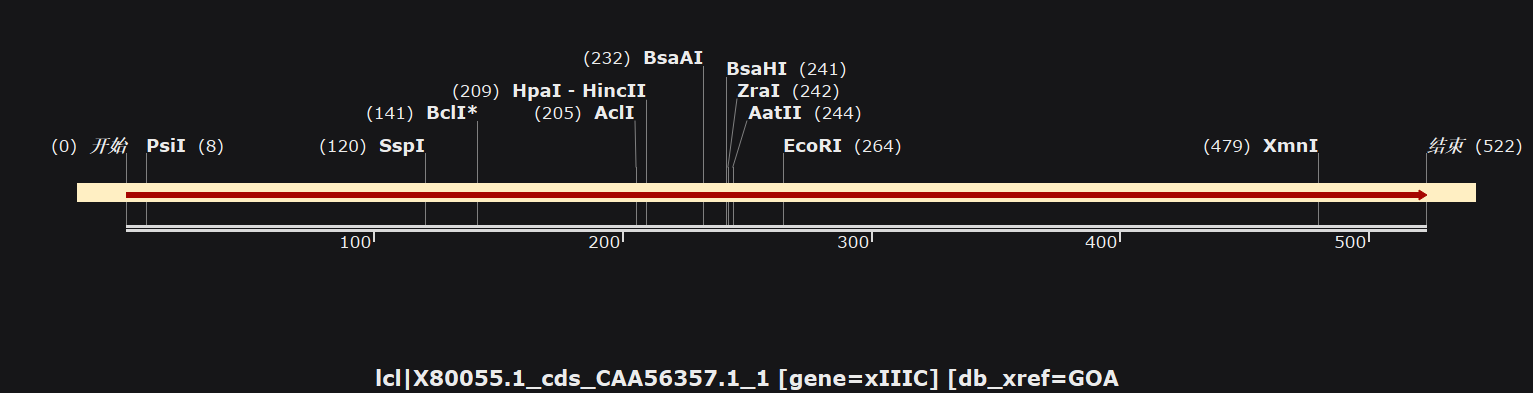


Figure S2. Sequence comparison between X80055.1 (*ApxIIIC*) and APPFJLYC01.

The sequence shown corresponds to X80055.1, which contains the complete *ApxIIIC* gene. The red arrow indicates the homologous region identified in APPFJLYC01, spanning the full length of the *ApxIIIC* gene and demonstrating the presence of an intact *ApxIIIC* gene in APPFJLYC01.

**Figure.S3**


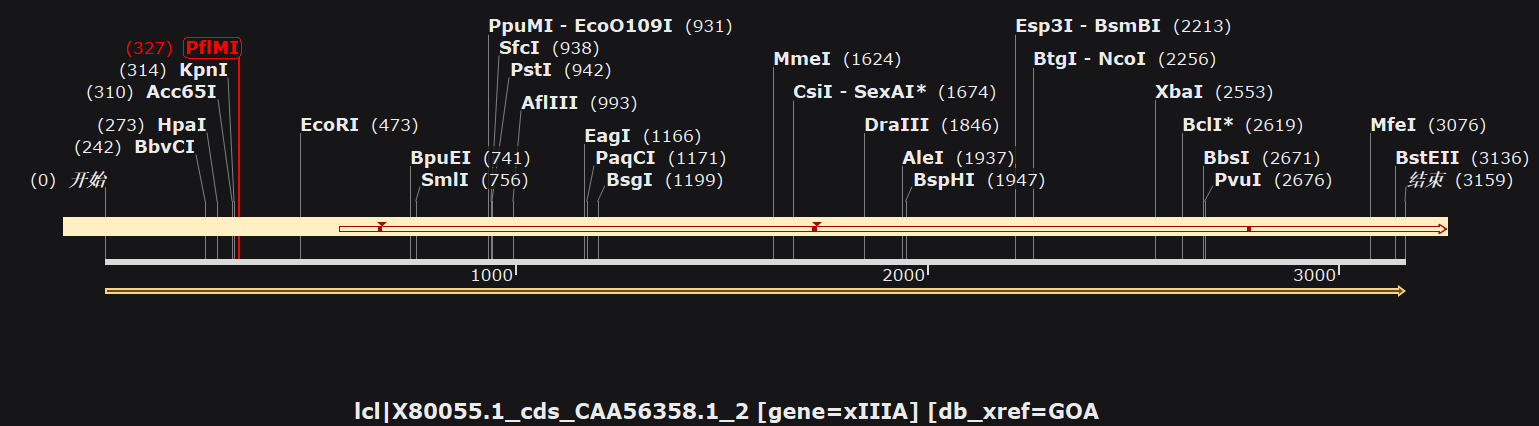


Figure S3. Sequence comparison between X80055.1 (*ApxIIIA*) and APPFJLYC01.

The sequence shown corresponds to X80055.1, which contains the complete *ApxIIIA* gene. The red arrow indicates the homologous region identified in APPFJLYC01, spanning the full length of the *ApxIIIA* gene sequence and confirming the presence of an intact *ApxIIIA* gene in APPFJLYC01.

**Figure.S4**


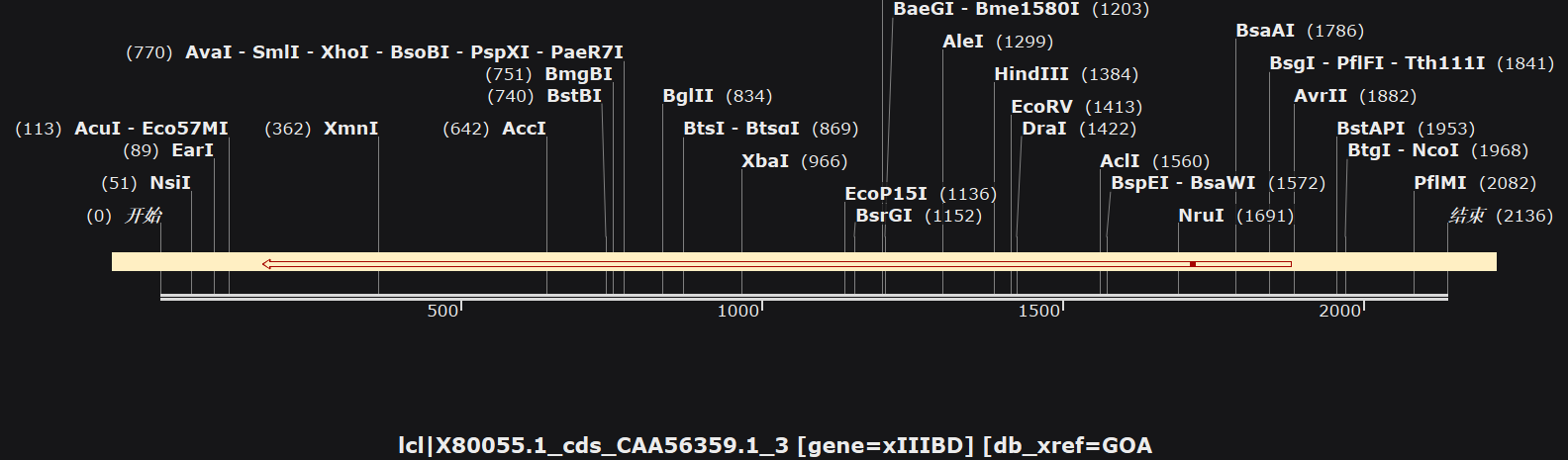


Figure S4. Comparative analysis of the *ApxIIIBD* region between X80055.1 and APPFJLYC01.

The red arrow denotes the homologous region shared by X80055.1 and APPFJLYC01, covering the entire *ApxIIIBD* gene region and indicating the presence of intact *ApxIIIBD* genes in APPFJLYC01.
